# Anaphylactic Degranulation of Mast Cells: Focus on Compound Exocytosis

**DOI:** 10.1155/2019/9542656

**Published:** 2019-03-18

**Authors:** Ofir Klein, Ronit Sagi-Eisenberg

**Affiliations:** Department of Cell and Developmental Biology, Sackler Faculty of Medicine, Tel Aviv University, Tel Aviv 69978, Israel

## Abstract

Anaphylaxis is a notorious type 2 immune response which may result in a systemic response and lead to death. A precondition for the unfolding of the anaphylactic shock is the secretion of inflammatory mediators from mast cells in response to an allergen, mostly through activation of the cells via the IgE-dependent pathway. While mast cells are specialized secretory cells that can secrete through a variety of exocytic modes, the most predominant mode exerted by the mast cell during anaphylaxis is compound exocytosis—a specialized form of regulated exocytosis where secretory granules fuse to one another. Here, we review the modes of regulated exocytosis in the mast cell and focus on compound exocytosis. We review historical landmarks in the research of compound exocytosis in mast cells and the methods available for investigating compound exocytosis. We also review the molecular mechanisms reported to underlie compound exocytosis in mast cells and expand further with reviewing key findings from other cell types. Finally, we discuss the possible reasons for the mast cell to utilize compound exocytosis during anaphylaxis, the conflicting evidence in different mast cell models, and the open questions in the field which remain to be answered.

## 1. Allergy and Anaphylaxis

Type 2 immune responses are tightly associated with allergy, a manifestation of clinical symptoms that are caused by hypersensitivity to food, insects, plants, or other airborne allergens. Severity of allergic reactions may range from local discomfort in cases such as a skin rash to death by anaphylaxis, defined by the World Health Organization as a severe, life threatening, generalized, or systemic hypersensitivity reaction [1]. The anaphylactic reaction is fast and can be triggered in various organs and tissues such as the skin, cardiac, gastrointestinal, and bronchopulmonary systems [2–5]. In lethal cases of anaphylactic shock, death may occur within an hour [6] and in some cases, even shorter than that [6, 7].

Key players in allergic reactions are mast cells (MCs) and basophils that by expressing the high affinity for immunoglobulin E (IgE) receptor (Fc*ε*RI) respond to allergen-specific IgE, whose formation is triggered by IL4 [8]. Following the crosslinking of cell-bound IgE molecules by their respective allergens and the consequent aggregation of the Fc*ε*RI, the MCs and basophils are activated effecting the robust release of a wide spectrum of inflammatory mediators [9–12]. Notably, though both basophils and MCs express the Fc*ε*RI, the IgE-dependent anaphylaxis reaction appears to be primarily mediated by MCs, as indicated by the failure of MC-deficient mice to develop an anaphylactic reaction in response to IgE/antigen [13]. Indeed, the blood level of tryptase, that is, exclusively released by activated MCs, is one of the most reliable diagnostic tests for anaphylaxis [4, 5, 14], and many cases of idiopathic anaphylaxis turn out to result from MC disorders [15]. Therefore, collectively, the evidence points to MCs as the key regulators of most forms of anaphylaxis.

MCs can also be activated independently of IgE by the complement system [16] or by a family of natural or synthetic basic secretagogues [17]. The latter were recently shown to activate MCs by binding to Mrgx2, a member of the family of G protein-coupled receptors (GPCRs) [17–20]. In humans, most of the IgE-independent anaphylactic responses are triggered by a variety of medications [4, 5, 16] and appear to rely on MC activation [21, 22]. Nonetheless, the majority of anaphylactic responses are IgE-dependent [23, 24].

## 2. Modes of Regulated Exocytosis in MCs

The hallmark of the allergic reaction is the immediate release by regulated exocytosis of inflammatory mediators that are presynthesized and prestored in MC secretory granules (SGs) [25]. The release process may occur by different mechanisms, including full exocytosis, kiss-and-run exocytosis, piecemeal degranulation, and compound exocytosis [26, 27].

**Figure 1:**
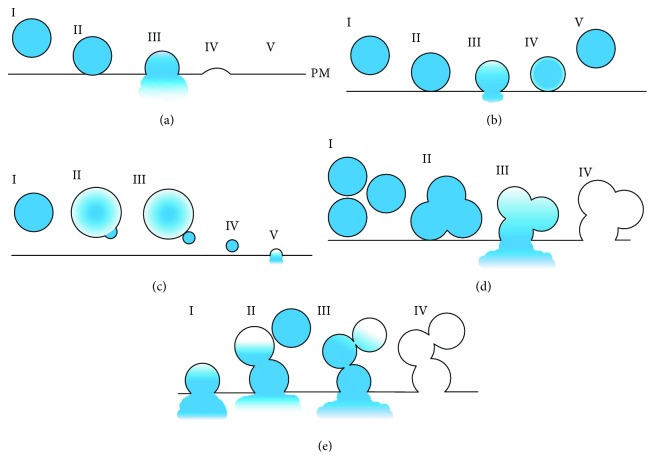
Modes of regulated exocytosis. A schematic presentation depicting the different modes of regulated exocytosis. (a) During full exocytosis, a SG (I) docks to the PM (II) and fuses with it to fully discharge all of its content (III), while fully collapsing into the PM (IV+V). (b) In kiss-and-run exocytosis, a SG (I) docks to the PM (II) and fuses with it (III) but does not collapse. Instead, the SG is retrieved back into the cytoplasm (IV+V). (c) During piecemeal degranulation, a “resting” SG (I) swells and packs a small amount of cargo into a budding vesicle (II). The small vesicle then buds off the “mother” SG (III) and is transported to the PM (IV) where it fully fuses with the PM and secretes its content to the extracellular milieu (V). (d) During compound exocytosis of a multigranular nature, several SGs fuse together to form a giant SG (I+II), which then fuses with the PM (III) to secrete its content, resulting in an empty degranulating sac (IV). (e) In compound exocytosis of a sequential nature, a single SG first fuses with the PM and begins secreting its content (I). However, the SG does not collapse into the PM, but instead a second SG fuses with the first one (II) and secretes its content through the primary SG which acts as a channel connecting to the extracellular milieu. This process continues with more SGs fusing with the growing channel (III) until secretion ends and all secretory cargo is released, resulting in an empty degranulating sac (IV).

During full exocytosis, which was first described in 1950 [28], a single secretory vesicle fully fuses with the plasma membrane (PM), resulting in the collapse of the SG and release of the SG content to the extracellular milieu (Figure 1(a)). In this form of exocytosis, homeostasis of the cell shape and its size is achieved by the tight coupling of the exocytic events with endocytosis of the PM that takes place in areas distal to the fusion site and is followed by recycling of the membrane to generate new secretory vesicles [29, 30].

Kiss-and-run exocytosis was suggested in the 1970s, as an alternative mode of exocytosis [31–33], during which a single secretory vesicle fuses with the PM, but does not collapse. Instead, a transient fusion pore is formed at the PM, which subsequently closes by the pinching off of the SG back into the cytoplasm (Figure 1(b)) allowing secretion of small cargo such as amines [34, 35], without exchange of lipids or proteins between the SG and the plasma membranes, while retaining a constant omega shape throughout the exocytic process. A more recently discovered close relative of kiss-and-run is cavicapture. Sometimes mistaken for kiss-and-run, during cavicapture, a transient fusion pore is also formed and an omega shape is retained. However, the fusion pore dilates to allow secretion of larger cargo, such as small proteins, and partial exchange of membrane components between the SG and the PM before the pinching off of the SG [36–44]. In comparison to full exocytosis, kiss-and-run exocytosis and cavicapture exocytosis offer a faster and more effective mechanism for granule recycling. Importantly, the fact that secretion takes place through a transient fusion pore of a definitive size implies that this mechanism may serve to restrict the size or limit the amount of cargo that can be released.

Piecemeal degranulation, a relatively rare form of regulated degranulation, was identified in MCs [45] and suggested to involve release through intermediate vesicles without the need of SG transport to the cell cortex and SG fusion with the PM. A mechanism, termed “the shuttling vesicle,” was suggested, whereby the SG swells and portions of its content are packed into small vesicles that bud from the SG and traffic to the PM, fusing and releasing their content [46] (Figure 1(c)).

Finally, compound exocytosis refers to exocytic events that involve homotypic fusion of several SGs, prior to (multigranular) or after (sequential) SG fusion with the PM. In multigranular exocytosis, several SGs fuse together to establish a giant SG, which then fuses with the PM, resulting in robust discharge of the SG's content (Figure 1(d)). In sequential exocytosis, a single SG fuses with the PM, but before collapsing or retrieval, it serves as a hook for a second SG, which fuses with the first one and so on and so forth (Figure 1(e)). This form of exocytosis creates a channel through which SGs that are located distal from the PM can release their content bypassing the need of transport to the cell periphery [47, 48].

Though all of these modes of regulated exocytosis have been shown to occur in MCs [26, 45, 47–53], it has been argued that, *in vivo*, compound exocytosis is the predominant form of regulated exocytosis during the allergic response [45]. Recently, it was shown that the mode of exocytosis is dictated by the stimulus type. Hence, while IgE-independent secretion triggered by substance P, endothelin 1, C3a, or C5a occurs by full fusion exocytosis, the characteristic mode of IgE-dependent exocytosis of MCs is compound exocytosis [54]. Therefore, compound exocytosis appears to be the hallmark of the immediate MC response during type 2 immunity responses to IgE/antigen.

## 3. Methods for Monitoring Compound Exocytosis

The first indication for the existence of a compound mode of exocytosis was obtained during the 1960s, on the basis of analyses of transmission electron microscopy images of stimulated pancreatic acinar cells [55] and MCs [56–58]. The latter revealed altered morphology of the SGs, where some granules, of lower electron density, also appeared to be connected to the PM, suggesting their active involvement in content discharge; however, others were fused to each other, though not necessarily connected to the PM. These studies demonstrated the occurrence of homotypic SG fusion in MCs. However, since transmission electron microscopy reveals only a thin section of the cell, connection to the PM of the fused granules might have been missed in the analyzed section. Therefore, these fused ultrastructures could be interpreted as multigranular fusion that occurred independently of fusion with the PM or that the SGs have fused to each other following a primary fusion event with the PM. Subsequent studies by Röhlich et al. [47] that were based on the light and transmission electron microscopies of MCs that were activated by the synthetic basic secretagogue, compound 48/80, have demonstrated that the “intracellular cavities” which communicated with extracellular space grew overtime after cell activation and spread into the cell center. These observations thus drove the conclusion that regulated exocytosis in MCs involves sequential SG fusion. Notably, these early studies that employed rat peritoneal MCs as a model have all implicated compound exocytosis in mediating MC responses to c48/80, unlike the more recent studies, mentioned above [54], that identified full exocytosis as the mode of secretion of MCs that are activated independently of IgE. Possible reasons for this discrepancy will be discussed below.

In the 1980s, electrophysiological measurements became a new technology for monitoring exocytosis and distinguishing between its distinct modes. Capacitance measurements by patch clamp enabled to estimate the increase in size of the cell surface membrane and thereby differentiate between fusion events of a single granule and fusion of multigranular structures that would yield a larger increase in membrane size and its corresponding change in capacitance [59–62]. Amperometric measurements, using a carbon fiber microelectrode, placed closely to the cell, recorded electrochemical changes that occurred when an oxidizable cargo, such as serotonin, in the case of the MCs [34], was released during degranulation. Combining both measurements then allowed to correlate the fusion events with the actual amounts of cargo released and thereby define the mode of exocytosis. Application of such combined patch clamp measurements to peritoneal MCs derived from mice or rats revealed the homotypic fusion of the SGs that occurs both prior and sequential to fusion with the PM [48, 60] and the compensatory endocytic process of compound endocytosis [44].

Patch clamp and electron microscopy were the only methods available for tracking compound exocytosis for decades. However, recent developments in light microscopy and the use of fluorescent reporters led to the establishment of new methods that allow monitoring the dynamics and spatiotemporal features of the exocytic events, including compound exocytosis, by live cell microscopy. In general, these methods can be categorized into two groups: (I) the tracking of the diffusion of a fluorescent marker from the SG to the extracellular milieu [54, 63–65] or (II) the tracking of the diffusion of a dye from the extracellular milieu into the fusing SG. The first approach requires SG loading with a fluorescent dye, such as fluorescently labeled dextrans [54, 63, 64, 66] that are taken up by the cells and sorted to the SGs [67–70], or cell transfection with a fluorescent reporter that would be targeted to the SGs and released in a regulated fashion [63, 65]. SG reporters that have been used in this regard include fluorescent-CD63 that translocates from the SG to the PM during exocytosis [71], neuropeptide Y (NPY) fused to mRFP [63], and *β*-hexosaminidase-pHluorin [65]. The second approach relies on the addition of a dye to the cell culture media and tracking its penetration into the cell, through the fusion pore at the plasma membrane and into the SG [72, 73]. The advantage of this method is that it also allows to estimate the size of the fusion pore by using dyes of different globular sizes and defining the threshold size of the penetrating dye [51, 72, 74–76]. An alternative version to this approach is the use of fluorescent avidin that binds to glycoproteins that are exteriorized during exocytosis [77]. This method was recently shown to successfully track *in vivo* exocytosis of MCs in mice [54].

The choice of reporters for MC exocytosis needs to take into account the fact that MC SGs maintain an acidic pH [78–80]. Therefore, to be able to visualize the SGs, a fluorescent protein that is insensitive to low pH needs to be employed. Such is the case of NPY-mRFP that is being used for this purpose [63, 81]. Alternatively, the actual fusion events can be monitored by using a pH-sensitive dye or protein such as fluorescein isothiocyanate (FITC) or the green fluorescent protein (GFP) variants. In this approach, the dye or transfected reporter is quenched when inside the acidic SG. However, once a fusion pore is formed and the SG's lumen alkalinizes due to its exposure to the external milieu, the dye/reporter regains their fluorescence, thus emitting a fluorescent signal concomitantly to the formation of the fusion pore [66, 82]. Based on this principle, FITC-dextran and *β*-hexosaminidase-pHluorin have been recently used for monitoring exocytosis in MCs [54, 63–65]. Moreover, these methods allow to correlate exocytosis with spatiotemporal changes in desired proteins or Ca^2+^ oscillations [54, 63–66]. Recording the robustness and duration of fluorescent signals also allows to distinguish between the different modes of exocytosis. For example, compound exocytosis can be distinguished from other exocytic modes simply through recording only long-lasting secretory events by setting long time intervals between acquisitions [82]. These modern live cell imaging techniques mark a new era, where exocytic events can be monitored in real time alongside protein trafficking and signaling events that occur in the cell. Thereby, these new methodologies improve our abilities to elucidate the underlying mechanisms of exocytic events, including compound exocytosis and identifying the molecular machineries involved. An instrumental tool towards this goal was achieved during our recent screen of Rab GTPases for their functional and phenotypic impacts on MC exocytosis [81]. During this screen, we noticed that expression of a constitutively active mutant of Rab5 results in the formation of giant SGs [81]. Further analysis revealed that expression of the constitutively active mutant of Rab5 increased the SGs' size while reducing their number, and conversely, silencing of Rab5 has increased the SG number but reduced their size [83]. This converse relationship has identified Rab5 as a regulator of SG fusion during their biogenesis. Because the giant SGs formed in cells that express a constitutively active mutant of Rab5 are exocytosis competent, they provide a useful model system that is easy to visualize and quantify by microscopy and therefore offers opportunities to the mechanisms of SG fusion, as discussed below.

## 4. Underlying Molecular Mechanisms of Compound Exocytosis

MC degranulation has been extensively studied over the past decades, and many key signaling events and machineries have been discovered and thoroughly reviewed in the literature [26, 27, 84–90]. However, the specific fusion machineries or precise mechanisms that link MC signaling with the distinct modes of exocytosis are only beginning to clear up. In particular, elucidating the underlying mechanism of compound exocytosis is challenging due to two main reasons: first, studying secretion as readout does not allow the distinction between the fusion machineries that mediate granule-granule fusion and those responsible for granule-PM fusion; and second, perturbation of one mode of exocytosis may be compensated by the takeover of an alternative mode, thus leaving the overall secretion unaffected. Therefore, also in this respect, real-time imaging of the secretory process may be the ultimate methodology to address this question.

The first hypothesis, proposing a mechanism for compound exocytosis, was put forward by Alvarez de Toledo and Fernandez [48], who, based on their electrophysiological measurements, proposed a model according to which, following the contact between the SG and the PM, the properties of the SG membrane change due to either the integration of PM proteins or changes in lipid composition. These changes then prime the SG allowing the fusion of a second SG to the first one. Consistent with this notion was the demonstration that SNAP23, a PM-localized SNARE protein, translocates to the SGs upon stimulation of permeabilized rat peritoneal MCs with Ca^2+^ and GTP*γ*S, conditions that also stimulated compound exocytosis [91]. Notably, translocation of SNAP23 occurs also under conditions of low temperature and hence does not require SG fusion. However, activation of SNAP23 function requires its phosphorylation by I*κ*B kinase 2 (IKK*β*) [54, 63, 92]. Whether or not this phosphorylation requires prior SG fusion with the PM is presently unknown. We have shown that in RBL-2H3 cells, both a phosphomimetic mutant and a phosphodeficient mutant of SNAP23 reside at the SGs in the absence of a cell trigger [63]. Therefore, the cellular location of SNAP23 may depend on phosphorylation cycles, which in turn may depend on fusion with the PM. Another SNARE protein involved in compound exocytosis is VAMP8 that was implicated in mediating SG-SG fusion during compound exocytosis in pancreatic acinar cells [93]. Based on our results, VAMP8 acts downstream of Rab5 in mediating homotypic SG fusion in RBL-2H3 cells [83], which makes VAMP8 an attractive candidate for mediating granule-granule fusion during compound exocytosis also in MCs.

Which of the syntaxin proteins that are expressed in MCs is involved in compound exocytosis is still debatable. In resting RBL-2H3 cells, both SNAP23 and syntaxin 4 (stx4) localize to the PM, but only SNAP23 translocates to the SGs upon IgE/antigen triggering [94]. In contrast, stx3 localizes to the SGs [71, 95]. While these results may implicate stx4 in mediating SG fusion with the PM and stx3 in mediating homotypic SG fusion, coimmunoprecipitation studies demonstrated complex assembly between SNAP23 and stx4 and its dependence on IKK*β*-mediated phosphorylation of SNAP23 [54, 92, 96]. These results therefore support a role of stx4 in compound exocytosis. Moreover, the coassembly of VAMP8 [93] with this complex further supports this concept.

Recent data has pointed to the mammalian uncoordinated-18 (Munc18) proteins as important regulators of compound exocytosis [13, 97]. Also known as stx-binding proteins, Munc18 proteins have been shown to bind to syntaxins to promote their closed conformation. Nonetheless, Munc18 proteins appear to be essential for SNARE complex assembly by clasping two complementary SNAREs and preventing their diffusion across membranes (reviewed in [98]). In addition, this family of proteins directly participates in vesicle trafficking and fusion events (reviewed in [84]). MCs express Munc18-1, Munc18-2, and Munc18-3 [99, 100]. Munc18-2 has been shown to coimmunoprecipitate mainly with stx3 and to a lesser extent with stx2, while Munc18-3 coimmunoprecipitates with stx4 [99]. However, Gutierrez et al. have recently demonstrated that the knockout of Munc18-1 or Munc18-3 does not affect secretion by mouse peritoneal MCs or development of anaphylactic responses, while the Munc18-2 knockout almost completely abolished secretion and multigranular compartments within the cells, along with strongly hindering the development of a systemic anaphylactic response in the knockout mice [97]. In accordance with these observations, independent research by Wu et al. has failed to assign a role for Munc18-1 in MCs during anaphylactic responses *in vitro* or *in vivo* [101]. However, Bin et al. have shown a small inhibition of exocytosis in response to IgE/antigen in Munc18-1-knocked-down RBL-2H3 cells and an even stronger inhibition of secretion in a double knockdown of Munc18-1 and Munc18-2, implying a synergistic role for these proteins [102]. Indeed, Brochetta et al. reported that Munc18-2 acts independently but synergistically with stx3 in mediating microtubule-dependent transport of stx3-positive vesicles to the PM [71]. Taken together, these data suggest that Munc18-2 is essential for the secretion of anaphylactic factors from MCs, possibly contributing to SG-SG fusion by mediating SG transport along the microtubules.

Munc13 proteins also play an important role in SNARE configuration. Munc13-4 acts sequentially to Munc18 and has been shown to mediate the transition of stx proteins from a closed to an open conformation, leading to the proper SNARE assembly during vesicle priming [103–105]. Indeed, mutations in Munc13-4 lead to type 3 familial hemophagocytic lymphohistiocytosis—a disorder in which cytotoxic T cells' granules dock, but do not fuse with the PM [106]. Furthermore, Munc13-4 has also been shown to play a role in fusion of recycling with late endosomes in cytotoxic T cells, a step that is required for the formation of secretory vesicles [107]. MCs express both Munc13-2 and Munc13-4 [13, 108]. However, while the knockout of Munc13-4 inhibited anaphylactic shock in the knockout mice, as well as MC secretion and SG-SG fusion in the bone marrow and peritoneal MCs derived from these mice [13], Munc13-2 only slowed down the rate of secretion [13], suggesting that Munc13-4 is the essential player in compound exocytosis.

In RBL-2H3 cells, Woo et al. have shown that Munc13-4 functions as a Ca^2+^ sensor through its C2A and C2B domains [109]. A similar role of Munc13-4, as a Ca^2+^ sensor during SG tethering, has also been shown in platelets, which are known to secrete through compound exocytosis [110]. In MCs, the function of Munc13-4 is inhibited by the direct interaction of Munc13-4 with Rab37 [111]. Taken together, these data point to Munc13-4 as a regulator of anaphylaxis by regulating compound exocytosis and to Rab37 as an inhibitor of its function. In this context, it is interesting to note that compound exocytosis induced by Fc*ε*RI activation in MCs cultured from human peripheral blood requires continuous oscillations of high Ca^2+^ while activation by substance P, which results in noncompound exocytosis, requires a short Ca^2+^ burst [54]. These results are consistent with a role of a Ca^2+^ sensor in dictating the mode of exocytosis that will take place in activated cells.

Other intriguing candidates for regulating compound exocytosis are the secretory carrier membrane proteins (SCAMPs) SCAMP1 and SCAMP2 that have previously been implicated in regulated exocytosis in neuroendocrine PC12 cells and in MCs [112–117]. Both SCAMP1 and SCAMP2 have been implicated in the regulation of fusion pore closure in PC12 cells [114, 116, 117]. However, while SCAMP1 facilitates the closure of the fusion pore and thereby limits the extent of compound exocytosis, SCAMP2 is essential for the dilation of the fusion pore [114, 116, 117] and may thus be crucial for maintaining a long-lived fusion pore that is required for compound exocytosis [113, 115]. Notably, SCMAP2 is abundantly expressed in both pancreatic acinar cells and MCs [115, 118, 119], both of which utilize compound exocytosis.

Finally, by monitoring the dequenching of SG-loaded FITC-dextran and directly tracking compound exocytosis events in Fc*ε*RI-triggered RBL-2H3 cells, we have recently demonstrated that Rab5, which we have previously identified as a regulator of SG-SG fusion during their biogenesis [83], fulfills a similar function during compound exocytosis [63]. We have shown that silencing of Rab5 completely abolishes compound exocytosis, while a constitutively active Rab5 mutant acts synergistically with SNAP23 in enhancing this process [63]. The identification of Rab5 as a regulator of SG fusion now provides us with a useful tool for exploring key steps in this process by identifying the Rab5 effectors that mediate its functions.

## 5. Lessons from Other Cells: Open Questions in MCs

While differences in the exocytic machinery between cell types are common [120], some key aspects of compound exocytosis have not yet been addressed in MCs. Perhaps the most uncharacterized aspect of compound exocytosis concerns the differences between sequential exocytosis and multigranular exocytosis. In lactotrophs, protein kinase C (PKC) activation has been shown to be crucial for both the primary SG-PM fusion event and its following SG-SG fusion events of sequential exocytosis [121]. In contrast, cAMP signaling is required for the secondary fusion, revealing a possible mechanism for differentiating sequential exocytosis from multigranular exocytosis or full fusion exocytosis [121]. How precisely MC signaling couples to their different modes of exocytosis remains to be resolved.

In pancreatic acinar cells, homotypic fusion between zymogen granules was found to be markedly less sensitive to Ca^2+^ than fusion between the zymogen granules with the PM [122]. Similarly, Hartmann et al. found that in eosinophils, the rate of SG-PM fusion was sensitive to both GTP*γ*S and Ca^2+^, the rate of SG-SG fusion was sensitive to GTP*γ*S but insensitive to Ca^2+^, and the rate of fusion between a SG and a granule that is already fused to the PM was sensitive to Ca^2+^ but insensitive to GTP*γ*S [123]. These observations clearly point to different mechanisms that underlie granular fusion events that occur during multigranular exocytosis or sequential exocytosis and imply their regulation by distinct GTPases and/or Ca^2+^ sensors. Consistent with this notion, as already mentioned above, Gaudenzio et al. have shown that different modes of exocytosis in MCs are coupled to distinct patterns of Ca^2+^ mobilization [54]. However, which GTPases or Ca^2+^ sensors regulate MC exocytosis, remains to be resolved. As described above, so far, Rab5 and Munc13-4 have been recognized in this context; however, their precise coupling to stimulus-specific signaling events awaits future research.

Activation of eosinophils by concanavalin A induces SG-SG fusion but not exocytosis [123]. Thus, a similar mechanism may also apply to MCs, where pre-exposure to a specific milieu or trigger may shift the exocytic response to a predominant multigranular response when the appropriate exocytic signal arrives.

Finally, an intricate part of the secretory machinery is the actin cytoskeleton. During exocytosis, actin has been proposed to serve as a barrier for exocytosis [124–126], preventing fusion of the SG with the PM, but has also been shown to be crucial for SG transport and the squeezing of large or dense exocytic compartments by an actomyosin meshwork, to assist the expulsion of the SG's content (reviewed in [127]). Thus, since compound exocytosis relies on the secretion of large SGs, it is plausible that secretion of multigranular compartments would require the assistance of the actomyosin meshwork. Strikingly, growing evidence in the lacrimal gland cells [128], salivary glands [129, 130], and pancreatic acinar cells [131] reveals that inhibition of actomyosin assembly results in enhanced formation of large vesicles that are fused to the PM. Two possible mechanisms have been suggested to underlie this phenotype. The first model suggests that actin coats serve as a barrier to limit homotypic SG fusion, similar to the role of the cortical actin in limiting SG fusion with the PM. Such mechanism would require controlled removal of the actin coating, thus allowing sequential fusion and compound exocytosis to occur in a regulated manner [132–134]. The second model suggests that inhibition of actomyosin complex formation inhibits the compensatory endocytosis that follows exocytosis. Under such conditions, diffusion of proteins and lipids from the PM to the fused SG-primed sequential fusion events results in enlarged, i.e., fused SGs [93, 135]. Alternatively, actin structures, localized to the fusion pore, may stabilize the primary fusion pore and thereby prevent SG collapse, thus facilitating sequential exocytosis [125, 126]. In MCs, the role of actin in the formation or stabilization of multigranular vesicles has yet to be addressed.

## 6. Compound Exocytosis: What for?

Compound exocytosis allows the release of SGs that reside in the cell center without the need for their transport to the cell periphery [73]. It also allows the release of a substantial amount of cargo at once. Indeed, multigranular fusion will give rise to giant organelles that can store up to 4-fold more cargo than a single SG [51]. Based on these features, compound exocytosis has long been considered the most efficient and most massive form of regulated exocytosis. This concept undoubtedly applies to secretion by eosinophils and neutrophils. Both are parasite-killing cells, which also use both multigranular exocytosis and sequential exocytosis as a tool for targeted and robust release of antiparasitic agents [62, 123, 136–140]. MCs are also antiparasitic cells known to be important in helminth immunity [141–143]. They are also capable of targeted secretion [144] and may have therefore developed this mechanism for their host defense activities.

Compound exocytosis also provides a clear advantage when cells need to secrete into a small lumen, such as in the case of acinar cells [134]. Thus, rather than individually fusing with the PM, which would require a large surface area and an extended lumen to secrete to, release by compound exocytosis allows multiple granules to discharge their content through a single granule that has access to the lumen.

**Figure 2:**
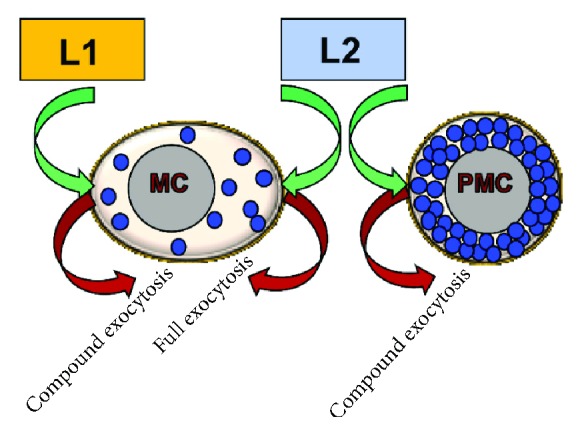
The mode of exocytosis is both stimulus- and MC type-dependent. A model is presented for the intricacy of the decision-making for choosing the mode of exocytosis that will take place in an activated MC. According to this model, the same MC may respond to one stimulus (L1) by compound exocytosis, whereas to another stimulus (L2) by full exocytosis. However, peritoneal MCs (PMC), whose cytoplasm is tightly packed with SGs, would respond by compound exocytosis also to ligands such as L2 that trigger full exocytosis in other MC types.

Finally, compound exocytosis also provides an important advantage when cells are fully packed with SGs. Hence, while fusion with the PM of multiple SGs will require extensive compensatory endocytosis to maintain cell size homeostasis, secretion through a multigranular channel will not affect the cell size if the giant structure simply pinches off. In fact, it has been suggested that the pinched-off, endocytosed degranulation sac may serve as a new efficient secretory organelle [145]. Relevant to this advantage of compound exocytosis might be the differences noted in the mode of exocytosis when comparing distinct MC types. Hence, as already alluded to earlier in this review, while early studies have documented compound exocytosis in compound 48/80-stimulated MCs [47, 48, 58], more recent studies have challenged this dogma showing that substance P, C3a, C5a, and ET1 that like compound 48/80 are considered basic secretagogues, do not stimulate this mode of release [54]. The trivial explanation to this discrepancy would be that the synthetic compound 48/80 induces secretion by a different mechanism than the physiological ligands. However, an alternative view would be that the mode of secretion is not only stimulus-dependent but also cell type-dependent (Figure 2). In this context, rat peritoneal MCs, which were used as a model in the earlier studies [47, 58], are packed with SGs and may therefore employ compound exocytosis for the reasons described above. Indeed, this notion is supported by studies by Balseiro-Gomez et al. who demonstrated that activation by corticotropin-releasing hormone (CRH) of mucosal MCs derived from the mouse intestine results in secretion of small SGs, in what appeared to be piecemeal degranulation, while activation of peritoneal MCs with the same agonist results in the formation of giant SGs and compound exocytosis [146]. Therefore, taken together, the evidence seems to suggest that compound exocytosis is a mechanism that serves the needs of the cell itself or its obligations to the cell environment.

## 7. Concluding Remarks

The mechanism by which MCs induce allergic responses, including the notorious anaphylactic shock, mainly involves secretion of preformed mediators through compound exocytosis. Although little is known about the signaling pathways that couple to the different modes of regulated exocytosis and even less on the signals that determine which type of compound exocytosis will take place, significant and exciting discoveries have recently been made that advance our understanding. However, we are still a long way from fully understanding the pathways leading to compound exocytosis, and fundamental questions in the field remain to be addressed. What is the physiological importance of compound exocytosis? What is the clinical significance of sequential secretion vs. multigranular secretion? Is there a difference in the type of cargo being secreted through different exocytic modes? What are the traits of the different MC populations that result in different exocytic responses to the same stimulus? With the development of new methods for tracking and manipulating compound exocytosis, newer approaches could now be utilized along with more traditional methods to further investigate these open questions.
